# Genome Sequences of Two Pseudomonas Isolates That Can Use Metformin as the Sole Nitrogen Source

**DOI:** 10.1128/mra.00639-22

**Published:** 2022-08-18

**Authors:** Katie B. Hillmann, Thomas D. Niehaus

**Affiliations:** a Department of Plant and Microbial Biology, University of Minnesota, St. Paul, Minnesota, USA; Wellesley College

## Abstract

Metformin is a major water pollutant globally. We report the complete genome sequences of two pseudomonads, Pseudomonas sp. strain KHPS1 and Pseudomonas hydrolytica strain KHPS2, isolated from wastewater treatment plant sludge, which can grow on metformin as the nitrogen source. Both isolates contained ~80-kb plasmids that may contain metformin breakdown genes.

## ANNOUNCEMENT

Metformin is one of the most prescribed medications in use today and has become the most prevalent anthropogenic surface water pollutant worldwide (1, 2). Isolation of metformin-metabolizing microbes and identification of the yet-unknown gene(s) involved in its breakdown will enable bioremediation strategies to be explored.

Primary sludge was collected from the Metropolitan Council Environmental Services Wastewater Treatment Plant in Minneapolis, MN, in October 2021. An aliquot of sludge (1 g) was added to 50 mL of citrate-acetate minimal medium containing 1 mM metformin as the only source of nitrogen. The culture was incubated at 37°C with shaking for 1 week, after which 0.5 mL was used to inoculate fresh medium and, as a control, medium lacking added nitrogen. The cultures were grown for an additional week. After three successive rounds of subculturing as above, only the cultures supplemented with metformin showed growth. An aliquot of culture (0.05 mL) was plated onto citrate-acetate minimal medium containing 1 mM metformin solidified with 15 g L^−1^ agar and incubated at 37°C overnight. Several colonies appeared on the plate; these were streaked onto solid LB medium to obtain single colonies and then grown again on minimal medium containing 1 mM metformin to confirm that each isolate represented a single species able to metabolize metformin and not a coculture. Full-length 16S rRNA genes from each isolate were PCR amplified and sequenced using the primers 1512uR and 8bF (3). 16S sequence analysis showed that our isolates consisted of at least two pseudomonads. Each was cultured overnight in 5 mL LB medium, and DNA was isolated using the GenElute bacterial genomic DNA kit (Sigma-Aldrich).

DNA was sent to the Microbial Genome Sequencing Center (https://www.migscenter.com/) for Illumina library preparation and sequencing and Oxford Nanopore Technologies sequencing. For Illumina sequencing, DNA was fragmented and sample libraries were prepared using the Illumina DNA prep kit and IDT 10-bp unique dual indices (UDIs) and sequenced on an Illumina NextSeq 2000 instrument (151-bp paired-end reads). Demultiplexing, quality control, and adapter trimming were performed using bcl-convert v3.9.3. For Nanopore sequencing, PCR-free ligation libraries were prepared and sequenced on a MinION_R9 flow cell. Guppy v4.2.2 (Oxford Nanopore) was used for base calling. Quality control and adapter trimming were performed using Porechop (https://github.com/rrwick/Porechop). *De novo* hybrid assembly and polishing of the Illumina and Nanopore reads were performed using Unicycler v4.9 with default parameters (4). Genomes were rotated to begin with the *dnaA* gene. Assembly annotation was performed using Prokka (5). Metrics for sequencing and assembly and a summary of the Pseudomonas genomes are listed in Table 1. One isolate was designated Pseudomonas sp. strain KHPS1 because its 16S rRNA sequence is 100% identical to that of Pseudomonas sp. strain A230. The genome of the other isolate has an average nucleotide identity (6) of 97.588% to the type genome of Pseudomonas hydrolytica, with 84.1% genome coverage, and was designated Pseudomonas hydrolytica strain KHPS2.

**TABLE 1:** Sequencing, assembly, and genome metrics

Metric	Data for strain:	
	Pseudomonas sp. KHPS1	Pseudomonas hydrolytica KHPS2
No. of ONT reads	248,489	416,048
No. of Illumina reads	3,167,247	1,765,333
Genome coverage (×)	195	99
*N*_50_ (bp)	5,061,778	5,414,044
GC content (%)	64.45	64.39
Genome size (bp)	5,061,778	5,414,044
Plasmid size (bp)	79,836	83,915

Since genes encoding new environmentally relevant enzymatic functions can be shared among a microbial community via plasmid (7), we predicted that metformin breakdown genes were located on the plasmid of each of our isolates.

### Data availability.

The GenBank accession numbers for the complete genome and plasmid are CP100551.1 and CP100552.1 for Pseudomonas sp. KHPS1 and CP100553.1 and CP100554.1 for Pseudomonas *hydrolytica* strain KHPS2, respectively. The raw sequence reads are available in the Sequence Read Archive under accession number PRJNA851084.

## References

[1] Wilkinson JL, Boxall ABA, Kolpin DW, Leung KMY, Lai RWS, Galbán-Malagón C, Adell AD, Mondon J, Metian M, Marchant RA, Bouzas-Monroy A, Cuni-Sanchez A, Coors A, Carriquiriborde P, Rojo M, Gordon C, Cara M, Moermond M, Luarte T, Petrosyan V, Perikhanyan Y, Mahon CS, McGurk CJ, Hofmann T, Kormoker T, Iniguez V, Guzman-Otazo J, Tavares JL, Gildasio De Figueiredo F, Razzolini MTP, Dougnon V, Gbaguidi G, Traoré O, Blais JM, Kimpe LE, Wong M, Wong D, Ntchantcho R, Pizarro J, Ying G-G, Chen C-E, Páez M, Martínez-Lara J, Otamonga J-P, Poté J, Ifo SA, Wilson P, Echeverría-Sáenz S, Udikovic-Kolic N, Milakovic M, et al. 2022. Pharmaceutical pollution of the world’s rivers. Proc Natl Acad Sci USA 119:e2113947119. doi:10.1073/pnas.2113947119. 35165193 PMC8872717

[2] Wang Y-W, He S-J, Feng X, Cheng J, Luo Y-T, Tian L, Huang Q. 2017. Metformin: a review of its potential indications. Drug Des Devel Ther 11:2421–2429. doi:10.2147/DDDT.S141675. PMC5574599 28860713

[3] Morillo V, Abreu F, Araujo AC, de Almeida LGP, Enrich-Prast A, Farina M, de Vasconcelos ATR, Bazylinski DA, Lins U. 2014. Isolation, cultivation and genomic analysis of magnetosome biomineralization genes of a new genus of South-seeking magnetotactic cocci within the Alphaproteobacteria. Front Microbiol 5:72. doi:10.3389/fmicb.2014.00072. 24616719 PMC3934378

[4] Wick RR, Judd LM, Gorrie CL, Holt KE. 2017. Unicycler: resolving bacterial genome assemblies from short and long sequencing reads. PLoS Comput Biol 13:e1005595. doi:10.1371/journal.pcbi.1005595. 28594827 PMC5481147

[5] Seemann T. 2014. Prokka: rapid prokaryotic genome annotation. Bioinformatics 30:2068–2069. doi:10.1093/bioinformatics/btu153. 24642063

[6] Ciufo S, Kannan S, Sharma S, Badretdin A, Clark K, Turner S, Brover S, Schoch CL, Kimchi A, DiCuccio M. 2018. Using average nucleotide identity to improve taxonomic assignments in prokaryotic genomes at the NCBI. Int J Syst Evol Microbiol 68:2386–2392. doi:10.1099/ijsem.0.002809. 29792589 PMC6978984

[7] Shintani M, Sanchez ZK, Kimbara K. 2015. Genomics of microbial plasmids: classification and identification based on replication and transfer systems and host taxonomy. Front Microbiol 6:242. doi:10.3389/fmicb.2015.00242. 25873913 PMC4379921

